# Non-Targeted Metabolomics Reveal Apomorphine’s Therapeutic Effects and Lysophospholipid Alterations in Steatohepatitis

**DOI:** 10.3390/antiox13111293

**Published:** 2024-10-25

**Authors:** Hideo Ogiso, Kouichi Miura, Ryozo Nagai, Hitoshi Osaka, Kenichi Aizawa

**Affiliations:** 1 Division of Clinical Pharmacology, Department of Pharmacology, Jichi Medical University, Shimotsuke 329-0498, Japan; 2 Division of Gastroenterology, Department of Medicine, Jichi Medical University, Shimotsuke 329-0498, Japan; 3 Jichi Medical University, Shimotsuke 329-0498, Japan; 4 Department of Pediatrics, Jichi Medical University, Shimotsuke 329-0498, Japan; 5 Clinical Pharmacology Center, Jichi Medical University Hospital, Shimotsuke 329-0498, Japan; 6 Division of Translational Research, Clinical Research Center, Jichi Medical University Hospital, Shimotsuke 329-0498, Japan

**Keywords:** metabolic dysfunction-associated steatohepatitis, non-alcoholic steatohepatitis, PTEN knockout, apomorphine, lysophospolipid, lysophosphatidylcholine, oxidative stress, liver disease, metabolomics, lipidomics

## Abstract

Metabolic dysfunction-associated steatohepatitis (MASH), characterized by progressive inflammation and fibrosis, evolves from metabolic dysfunction-associated steatotic liver disease and significantly heightens the risk of cirrhosis and hepatocellular carcinoma. Understanding metabolic pathways that influence MASH progression is crucial for developing targeted therapies. Non-targeted metabolomics offer a comprehensive view of metabolic alterations, enabling identification of novel biomarkers and pathways without preconceived ideas. Conversely, targeted metabolomics deliver precise and reproducible measurements, focusing on predefined metabolites to accurately quantify established pathways. This study utilized hepatocyte-specific PTEN knockout mice as a model to explore metabolic shifts associated with MASH. By integrating non-targeted metabolomics and targeted metabolomics, we analyzed liver samples from three groups: normal, pathological (MASH-affected), and MASH-affected, but treated with apomorphine, an antioxidant and recently reported ferroptosis inhibitor with potential therapeutic effects. Metabolic profiling identified lysophospholipids (LPLs) as significantly altered metabolites, with elevated levels in the MASH model and a notable reduction after apomorphine treatment. This suggests that LPLs are central to the etiology of MASH and may serve as targets for therapeutic intervention. Our findings underscore the effectiveness of apomorphine in modulating disease-specific metabolic disruptions, offering insights into its potential as a treatment for human MASH.

## 1. Introduction

Metabolic dysfunction-associated steatotic liver disease (MASLD, previously known as non-alcoholic fatty liver disease or NAFLD) frequently correlates with metabolic syndrome, characterized by excess lipid accumulation in hepatocytes leading to hepatic steatosis [1,2]. This condition can progress to metabolic dysfunction-associated steatohepatitis (MASH), marked not only by steatosis, but also by inflammation, hepatocyte injury, and fibrosis, significantly increasing the risk of cirrhosis and hepatocellular carcinoma [3,4]. Understanding these pathological transitions is crucial for early diagnosis and treatment development. Metabolite profiling, which has played a central role in identifying biomarkers for MASH, utilizes distinct metabolic signatures associated with this disease to improve diagnosis and monitor therapeutic efficacy [5,6,7,8,9,10,11,12]. This approach is essential, as it addresses the need for reliable diagnostic tools that can detect MASH at its onset, potentially guiding early therapeutic intervention.

Several dietary and genetic murine models of MASH have been extensively used to study MASH, yet few have replicated all metabolic, histological, and genetic features of the human condition [13,14,15]. Among these, hepatocyte-specific, PTEN knockout (PTEN KO) mice reportedly exhibit MASH-like pathological features, such as hepatic lipidosis, pericellular fibrosis, the presence of inflammatory cells, and subsequent carcinogenesis [16,17]. Additionally, PTEN protein expression is downregulated in some human hepatocellular carcinoma (HCC) cases [18].

Apomorphine, a non-selective dopamine agonist, is currently used to treat Parkinson’s disease. In light of apomorphine’s significant antioxidant properties, which were previously documented by our team [19], this study utilized apomorphine as a therapeutic agent to explore its potential effects on liver pathology in the PTEN KO model of MASH. This decision was grounded in our prior findings that demonstrated apomorphine’s efficacy in reducing liver steatosis and inflammation, symptoms central to MASH pathology. The chemical structure of apomorphine, C_17_H_17_NO_2_·HCl·0.5H_2_O, allows it to interact with dopaminergic receptors, and it has been historically used to manage “off” episodes in Parkinson’s disease patients.

In this study, using liquid chromatography-mass spectrometry, we analyzed liver tissue from PTEN KO mice, employed as a MASH model, to compare metabolic profiles among three groups: normal, pathological, and treated. This approach enabled identification of metabolites that characterize the pathological and the post-treatment recovery states. Based on our prior studies, apomorphine (Apo) was utilized as the treatment agent [19]. The present results demonstrated that certain liver metabolites, which were elevated in the pathological group, decreased in response to Apo administration.

## 2. Materials and Methods

### 2.1. Chemicals and Reagents

Methanol, acetonitrile, 2-propanol, ultra-pure water, and formic acid were of LC-MS grade. Butanol and 1 mol/L ammonium formate were HPLC grade. Ethyl acetate, hexane, and disodium hydrogen phosphate were special grade. Apo was biochemical grade. These reagents were purchased from FUJIFILM Wako Pure Chemical (Osaka, Japan). Potassium dihydrogen phosphate was special grade from Kanto Chemical (Tokyo, Japan). Dulbecco’s phosphate buffered saline (PBS) was purchased from Nacalai Tesque (Kyoto, Japan).

### 2.2. Animal Experiments

Hepatocyte-specific PTEN knockout (PTEN KO) mice and control mice, Albumin-Cre recombinase-negative PTEN flox/flox mice (littermates), were generated as previously reported [16]. All mice had a C57Bl6 background and 8-week old male mice were used. Mice were fed standard chow (MFG-LID, Oriental Yeast Co., Ltd., Tokyo, Japan) and had ad libitum access to food and water until the end of the experiments, under specific pathogen-free conditions. Ten mice were used in each group, for a total of 30 mice.

Apomorphine, dissolved in 3% DMSO, was injected daily (intraperitoneally) for 14 days. After 2 weeks of treatment, mice were killed, and harvested samples were stored at −80 °C, until use. Apomorphine was chosen as the therapeutic agent due to its previously demonstrated efficacy in modulating inflammatory pathways and reducing fibrosis in hepatocyte-specific PTEN knockout mice [19].

Animal experiments were approved by the Review Board of Jichi Medical University (permission number 20061; Tochigi, Japan). All animals received humane care according to criteria outlined in the *Guide for the Care and Use of Laboratory Animals*, published by the National Academy of Science, as well as the policies of Jichi Medical University.

For histological analysis, liver tissue was fixed in formalin, embedded in paraffin, and sectioned at a thickness of 4 µm. Sections were stained with hematoxylin and eosin (H&E) and subjected to histological examination using a Keyence microscope (Keyence, Tokyo). NAFLD activity scoring was conducted on ten randomly selected fields for each group, following published protocols [20,21]. Blood samples were collected from the inferior vena cava, and after centrifugation, the serum was analyzed. Serum aspartate aminotransferase (AST) and alanine aminotransferase (ALT) levels were assessed using FUJI DRI-CHEM SLIDE (FUJIFILM, Osaka, Japan) according to the manufacturer’s instructions. 

### 2.3. Sample Preparation for Metabolomics

Thirty micrograms of liver (wet weight) were homogenized with 300 µL of ice-cold PBS in 1.5 mL polypropylene vials. Then, 30 µL of 500 mM phosphate buffer (pH 5.8) and 750 µL of butanol were added to each sample. After vigorous shaking for 5 min, samples were centrifuged (12,000× *g* for 5 min at 4 °C), after which 600 µL of the upper layer were collected in 2 mL polypropylene vials. Original suspensions were re-extracted by adding 350 µL of ethyl acetate and 350 µL of hexane, followed by centrifugation. Second extracts (700 µL) were combined with the first butanol extracts, and then evaporated using a centrifugal evaporator for 90 min at 50 °C (Taitec VC-36S, Taitec, Saitama, Japan). Resultant dry materials were re-dissolved in 300 µL of methanol/2-propanol (1:1, *v*/*v*), and stored at −80 °C until use. For non-targeted metabolomic measurements, just before measurement, 20 μL of liver extracts were mixed with 180 μL of methanol/2-propanol (1:1), i.e., diluted 10-fold. Extracts were then centrifuged (12,000× *g* for 5 min at 4 °C), after which the upper layers were collected in 0.3 mL polypropylene vials. For targeted lipidomic measurements, extracts were diluted 100-fold with methanol/2-propanol (1:1), and centrifuged in the same manner to obtain supernatants. For analyte samples that saturated the MS detector at a 100-fold dilution, a 2000-fold dilution was used instead.

Protein amounts in residues after the second extraction were used to normalize detected amounts from MS analysis. Lipids were washed from each residue with a mixture of 700 µL of methanol and 700 µL of 2-propanol with sonication. After centrifugation (12,000× *g* for 5 min) to remove supernatants, 300 µL of ultra-pure water were added to precipitates. Samples were sonicated again to disperse insoluble materials. Then, 900 µL of 8 M guanidine hydrochloride were added to suspensions, and proteins were solubilized with incubation for 30 min at 60 °C. After centrifugation (12,000× *g* for 5 min), solubilized protein solutions were collected. Protein concentrations were determined using a BCA protein assay kit (Pierce, Rockford, IL, USA).

### 2.4. Non-Targeted Metabolomics Analysis Using LC-QTOF-MS

Liquid chromatography quadrupole time-of-flight mass spectrometry (LC-QTOF-MS) was performed on an LCMS9030 system (Shimadzu, Kyoto, Japan). Metabolites in liver extracts were separated on a YMC-Accura Triart C_18_ column (100 mm × 2.1 mm, 1.9 µm; YMC Corporation, Kyoto, Japan). Mobile phases A and B consisted of 0.1% (*v*/*v*) formic acid in ultra-pure water and 0.1% (*v*/*v*) formic acid in 1:1 acetonitrile/methanol, respectively. The initial condition was set at 0% B. The following solvent gradient was applied: 0% B for 1 min was followed by a linear gradient to 5% B from 1 to 3 min, to 90% B from 3 to 7 min, to 100% B from 7 to 11 min, and then held at 100% B from 11 to 15 min. Subsequently, the mobile phase was returned to the initial condition over 0.5 min, and was maintained for 4.5 min until the end of the run. The oven temperature was 45 °C and the flow rate was 0.32 mL/min. The sample volume injected was 1 μL.

Mass spectrometry (MS) data were acquired individually in positive (pos) and negative (neg) ion modes over 70 to 900 *m*/*z* using electrospray ionization. MS parameters used default settings as follows: interface voltage of 4.5 kV (pos) and −3.5 kV (neg), interface temperature of 300 °C, nebulization gas flow of 3 L/min, heating gas flow of 10 L/min, drying gas flow of 3 L/min, heat block temperature of 400 °C, and DL temperature of 250 °C. Before sample measurements, m/z values were calibrated using a calibrant (ESI Tuning Mix for Ion Trap, Supelco, Bellefonte, PA, USA), so that mass accuracy throughout sample measurements remained within 10 ppm. One representative among samples tested was used as a quality control (QC) sample. The QC sample was injected five times in total at the beginning, middle, and end of the batch to check stability of the detected amounts during measurements. Relative abundance of each component was evaluated by the peak height of the parent ion (MS1) in a full-scan mode. Additional measurements were also made in data-dependent, auto MS/MS mode to obtain fragmentation ions (MS2) necessary for structure estimation. Fragmentation was performed using argon as a collision gas at a collision energy of 30 eV with a spread of 15 eV. MS2 spectra were generated simultaneously for the top five ions with an *m*/*z* range between 50 and 900, surpassing an intensity threshold of 2000. Data were collected using LabSolutions software version 5.118 for the LCMS9030 (Shimadzu).

### 2.5. Data Processing and Statistical Analysis

Raw data (the mzML format) were processed using MS-DIAL version 5.1.230129 (http://prime.psc.riken.jp/compms/msdial/main.html, accessed on 20 February 2023) for peak detection, alignment and integration [22]. We tried to identify components from the exact mass values of elution peaks, with their elution times as complementary information. Searching the database (“ESI(+)-MS/MS from authentic standards” and “ESI(−)-MS/MS from authentic standards” containing information on 16,481 and 9,033 unique compounds, respectively), compound names were assigned based on MS1 exact mass values and MS2 spectra. MS-FINDER version 3.56 was also used to assist with assignments of compounds (http://prime.psc.riken.jp/compms/msfinder/main.html, accessed on 1 March 2023) [23]. A private list of priority compounds was used in conjunction with these databases. Sparse partial least squares-discriminant analysis (sPLS-DA) was performed with MetaboAnalyst version 6.0 (http://www.metaboanalyst.ca, accessed on 29 November 2023). Using MetaboAnalyst, analysis of variance (ANOVA) was performed among multiple groups, and box-plots were generated. Non-targeted metabolomics and extraction of characteristic components were performed by referring to previous reports [24,25,26].

### 2.6. Targeted Lipidomics Analysis Using LC-MS/MS

Liquid chromatography tandem quadrupole mass spectrometry (LC-MS/MS) analysis was performed on an LCMS8060 system (Shimadzu, Kyoto, Japan). Lipids were separated on a Shim-pack GISS C_18_ column (100 mm × 2.1 mm, 1.9 μm; Shimadzu). Mobile phases A and B consisted of 2-propanol/methanol/ultra-pure water/1 mol/L ammonium formate/formic acid (150:30:120:3:0.15, *v*/*v*/*v*/*v*/*v*) and (270:30:0:3:0.15, *v*/*v*/*v*/*v*/*v*), respectively. The initial condition was set at 0% B. The following solvent gradient was applied: 0% B was followed by a linear gradient to 50% B from 0 to 2 min, to 85% B from 2 to 11 min, to 100% B from 11 to 11.5 min, and then held from 11.5 to 15 min. Subsequently, the mobile phase was returned to the initial condition over 0.5 min and was maintained for 4.5 min until the end of the run. The oven temperature was 50 °C, and the flow rate was 0.2 mL/min. The sample volume injected was either 0.2 µL or 0.4 µL.

Targeted analytes were detected in multi-reaction monitoring (MRM) mode using electrospray ionization. MS parameters were used with default settings as follows: interface voltage of 4.0 kV (pos) and −3.0 kV (neg), nebulization gas flow of 1.5 L/min, heating gas flow of 5 L/min, drying gas flow of 10 L/min, heat block temperature of 400 °C, and DL temperature of 250 °C. Mass transitions and parameters are shown in Table S1. Data were collected using LabSolutions software version 5.123 for LCMS8060 (Shimadzu). The relative abundance of each component was evaluated by its peak area.

## 3. Results

### 3.1. Characteristics of PTEN KO Mice and Their Response to Treatment with Apo

**Figure 1:**
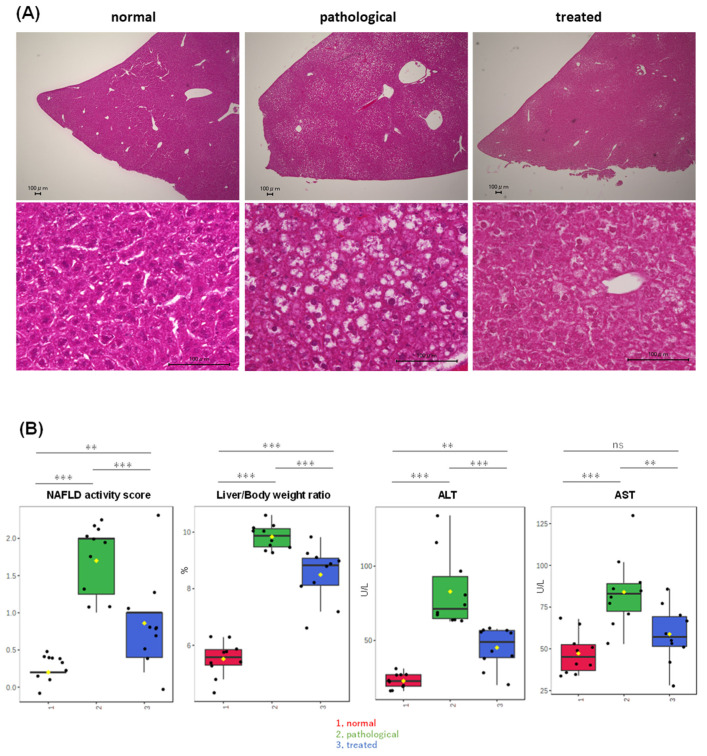
Partial improvement of adiposity and serum transaminase levels in PTEN KO mice by Apo administration. (**A**) Representative photos of H&E staining of the liver are shown. Control, PTEN KO, and Apo-treated PTEN KO mice are represented as normal, pathological, and treated states, respectively. (**B**) Comparison of MASH-related parameters across three groups, highlighting significant differences among three groups: (1, red) normal, (2, green) pathological, and (3, blue) treated. Yellow dots on the box and whiskers diagram represent the mean value and black dots indicate data points. ** *p* < 0.01, *** *p* < 0.001; ns, not significant.

PTEN KO mice at 10 weeks of age displayed moderate steatosis (Figure 1A), with minimal inflammatory cell infiltration and hepatocyte ballooning, indicating that PTEN KO mice at this age were in an early stage of MASLD. Treatment with apomorphine for two weeks significantly reduced steatosis severity (Figure 1A) and improved the NAFLD activity score, liver/body weight ratio, and serum transaminase levels (Figure 1B). In addition, apomorphine treatment decreased the NAFLD activity score, decreased the liver/body weight ratio, and reduced serum transaminase levels (Figure 1B).

### 3.2. Non-Targeted Metabolomic Analysis of Liver Extracts with LC-QTOF-MS

In terms of molecular pathogenesis of human MASH, the “multiple parallel hit theory” has generally been assumed [27,28,29]. In short, MASH is the result of numerous conditions acting in parallel, including abnormal lipid metabolism, oxidative stress, lipotoxicity, altered production of cytokines, gut dysbiosis, mitochondrial dysfunction, and other factors [30]. Before starting this study, we assumed that oxidized lipids contribute to pathogenesis of the MASH model. Therefore, for a comprehensive analysis of liver metabolites, liver extracts were prepared using a two-step extraction method. The first extraction with butanol was followed by a second extraction with a hexane/ethyl acetate mixture to extract a wide range of metabolites, such as short fatty acids and triglycerides. Second extracts were then used for measurement [31].

**Figure 2:**
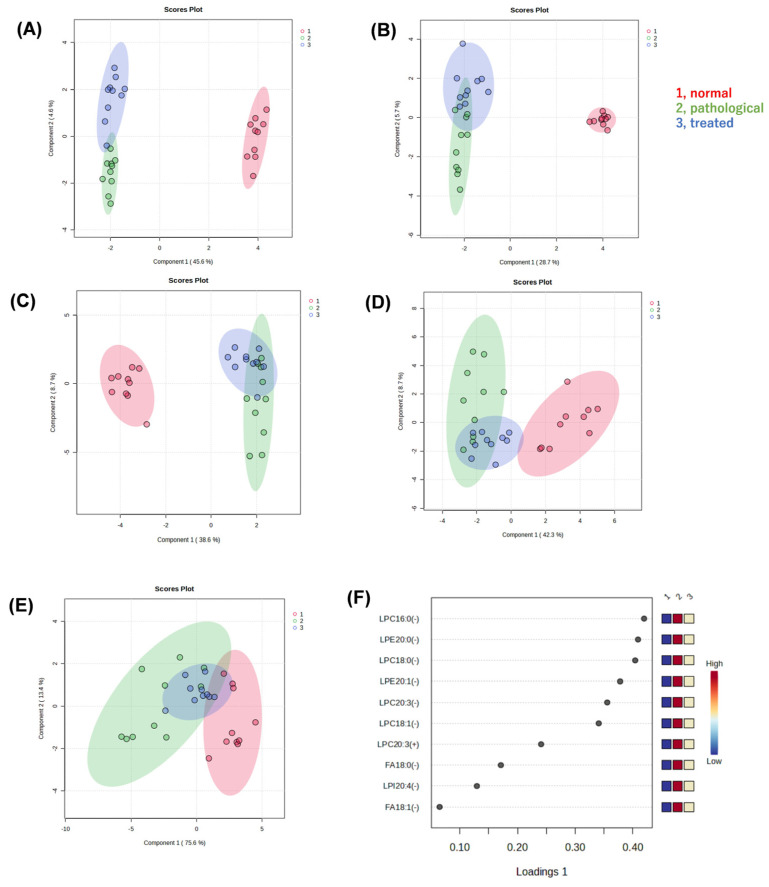
Multivariate statistical analysis of liver metabolite data generated by non-targeted metabolomics. (**A**) Score plot of a sparse partial least squares-discriminant analysis (sPLS-DA) with positive (**A**,**C**) or negative (**B**,**D**) ion data. Liver extracts from normal, pathological, and treated mice were analyzed by LC-QTOF-MS. All peaks detected were submitted for sPLS-DA (**A**,**B**). Only peaks that were searched against the metabolite databases and then assigned to known compounds were submitted to sPLS-DA (**C**,**D**). Furthermore, only peaks that were confirmed as lysophospholipids (LPLs) or fatty acids by their MS2 spectra and retention times were submitted to sPLS-DA. The resulting score plot (**E**) and loading plot (**F**) are shown. LPC, lysophosphatidylcholine; LPE, lysophosphatidylethanolamine; LPI, lysophosphatidylinositol; FA, fatty acid; 16:0, palmitic acid; 18:0, stearic acid; 18:1, oleic acid; 20:0, eicosanoic acid; 20:1, eicosenoic acid; 20:3, eicosatrienoic acid; 20:4, arachidonic acid.

We performed non-targeted metabolomic analysis of liver extracts of normal, pathological, and treated mice, using liquid chromatography quadrupole time-of-flight mass spectrometry (LC-QTOF-MS), to reveal metabolites associated with pathology and responses to treatment. In the resulting dataset, approximately 6300 peaks were detected in positive mode and 750 peaks in negative mode. After a database search, we identified 430 metabolites in positive mode and 150 metabolites in negative mode. Sparse partial least squares-discriminant analysis (sPLS-DA) was applied to determine whether there were differences in metabolic profiles among three groups, i.e., normal (vehicle-administated PTEN flox/flox), pathological (vehicle-administrated PTEN KO), and treated groups (Apo-administated PTEN KO). Clustering and separation among liver metabolites were evident in these three groups. When analyzing all data acquired, regardless of whether any compounds in the databases were assigned, the sPLS-DA model showed a separation trend among these three groups in positive (Figure 2A) and negative (Figure 2B) modes. We focused on metabolites associated with recovery from a pathological state to an ameliorated state with Apo treatment. Thus, the effect of Apo treatment on metabolites was reflected in component 2 of the score plot, but all peaks were unassigned, i.e., including unknown compounds or unidentified signals.

**Figure 3:**
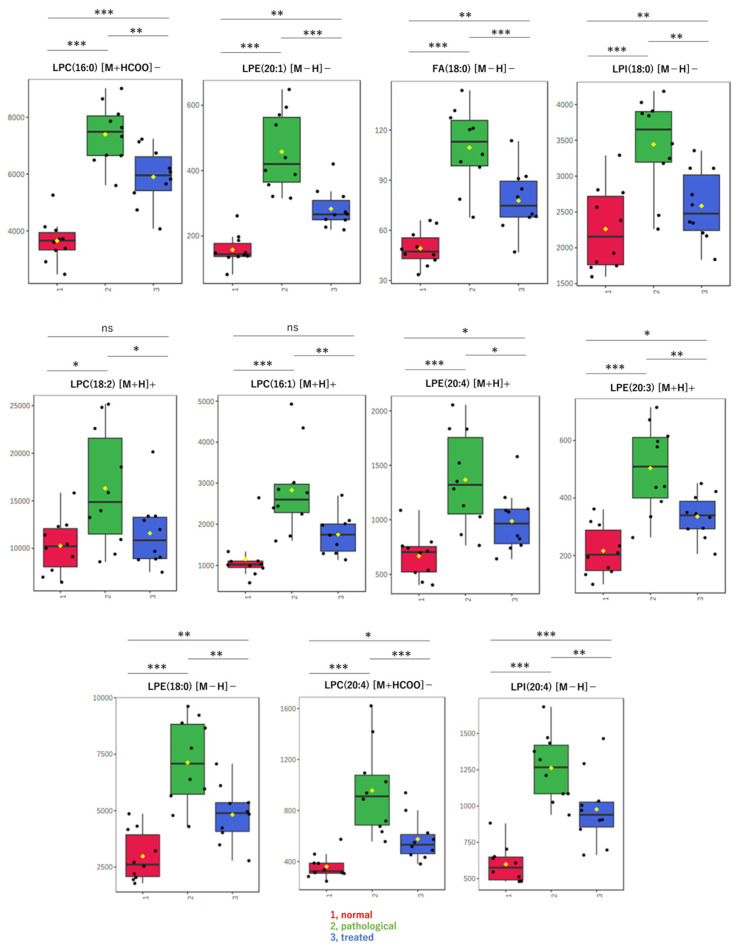
Box-plots of LPLs and fatty acids in liver extracts. These metabolites were evaluated as significant on multivariate analysis of non-targeted metabolomics. Detected amounts (peak heights) normalized by the protein amount were compared among three groups: (1, red) normal, (2, green) pathological, and (3, blue) treated. Yellow dots on the box and whiskers diagram represent the mean value and black dots indicate data points. * *p* < 0.05, ** *p* < 0.01, *** *p* < 0.001; ns, not significant; LPC, lysophosphatidylcholine; LPE, lysophosphatidylethanolamine; LPI, lysophosphatidylinositol; FA, fatty acid; 16:0, palmitic acid; 16:1, palmitoleic acid; 18:0, stearic acid; 18:2, linoleic acid; 20:3, eicosatrienoic acid; 20:4, arachidonic acid; [M + H]^+^, protonated ion; [M − H]^−^, deprotonated ion; [M + HCOO]^−^, formate adduct ion.

Next, when only data for known compounds in the databases were analyzed, whether correct or incorrect, the sPLS-DA model also showed a separation trend in positive (Figure 2C) and negative modes (Figure 2D). Also in the second analysis, the effect of Apo treatment on metabolites was reflected in component 2 of the score plot, most of which pertained to lysophospholipids (LPL) and fatty acids (FA). Using the sPLS-DA model, further analysis of data restricted to molecular species, such as lysophosphatidylcholine (LPC), lysophosphatidylethanolamine (LPE), lysophosphatidylinositol (LPI), and FA, reveals segregation trends (Figure 2E). The loading plot at that time is also shown (Figure 2F). Box-plots are provided for the top metabolites in the loading plot (Figure 3). All metabolites shown here increased in the pathological state and decreased toward normal values after treatment.

### 3.3. Targeted Lipidomic Analysis of Liver Extracts by LC-MS/MS

**Figure 4:**
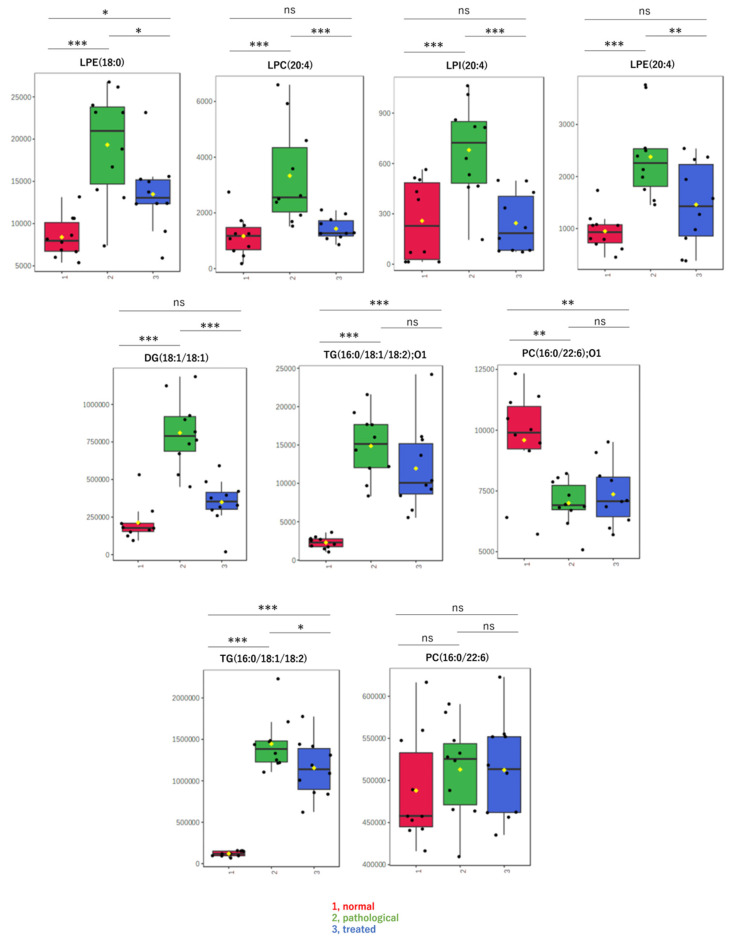
Box-plots of LPLs and other metabolites measured by targeted analysis. Detected amounts (peak areas) normalized by the protein amount were compared among three groups: (1, red) normal, (2, green) pathological, and (3, blue) treated. Yellow dots on the box and whiskers diagram represent the mean value and black dots indicate data points. * *p* < 0.05, ** *p* < 0.01, *** *p* < 0.001; ns, not significant; LPC, lysophosphatidylcholine; LPE, lysophosphatidylethanolamine; LPI, lysophosphatidylinositol; FA, fatty acid; DG, diacylglycerol; TG, triacylglycerol; 16:0, palmitic acid; 18:0, stearic acid; 18:1, oleic acid; 18:2, linoleic acid; 20:3, eicosatrienoic acid; 20:4, arachidonic acid; 22:6, docosahexaenoic acid; O1, mono-oxidized form.

By non-targeted metabolomic analysis, we determined that LPL and fatty acid species that change levels in a pathological state recovered to nearly normal levels following Apo treatment. Contrary to our expectations, oxidized lipids did not exhibit such changes. Therefore, to confirm results from non-targeted metabolomics and to explore other lipids, levels of specific molecular species, including LPLs, diacylglycerol, and oxidized lipids, were measured in a targeted analysis using LC-MS/MS. Box-plots are shown for lipids with relatively high detection intensities among the LC-MS/MS data, i.e., major components (Figure 4). The targeted analysis of representative LPLs yielded results similar to those of the non-targeted analysis, with increases in the pathological state and decreases to near-normal values after treatment. In addition, a diacylglycerol, DG(18:1/18:1), showed behavior similar to that of these LPLs. On the other hand, oxidized lipids, such as oxidized phosphatidyl choline “PC(16:0/22:6);O1” or oxidized triacylglycerol “TG(16:0/18:1/18:2);O1”, showed different behavior and almost no correlation with pathology. There were no significant differences in levels of these oxidized lipids between the disease and treatment groups (Figure 4).

## 4. Discussion

Globally, extensive research is ongoing to decipher molecular mechanisms underlying MASH, and to develop effective therapeutic agents [28,30,32]. MASH typically manifests as inflammation and fibrosis following excessive fat accumulation in the liver. Interestingly, the level of hepatic fat does not consistently correlate with the severity of inflammation and fibrosis [33]. Pathogenesis of human MASH has been described by theories such as the “two-hit theory” and the “multiple-parallel-hits theory” [27,28]. The “two-hit theory” suggests that oxidative stress or endotoxin exposure following fat deposition leads to inflammation, with persistent oxidative stress potentially driving the progression to steatohepatitis and hepatocellular carcinoma (HCC).

Numerous animal models have been employed to explore pathogenic mechanisms and potential treatments for MASH. These models include obesity-induced dietary models, nutrient-deficient dietary models, chemical-induced models, and genetically engineered models [13,14,15]. Despite this variety, few models fully replicate the complex metabolic, histological, and genetic features of the human disease. Among the genetic models, hepatocyte-specific PTEN KO mice develop features such as steatohepatitis, liver fibrosis, and subsequent carcinogenesis, closely mimicking the progression of human MASH, but with unique variations in pathogenesis, such as increased insulin sensitivity without the typical obesity-associated pathophysiology [16,17].

In this study, we focused on antioxidant properties of apomorphine [34,35,36,37], extensively documented by our team [19], to evaluate its therapeutic effects on MASH. Previous research has established apomorphine’s potential in ameliorating hepatic steatosis and inflammation, crucial elements of MASH pathology. Using liquid chromatography-mass spectrometry, we analyzed liver metabolites from PTEN KO mice that were used as a model for MASH. We compared metabolic profiles across three experimental groups: normal, pathological, and treated. This analysis revealed that metabolites, such as lysophospholipids (LPLs) and fatty acids, elevated in the pathological group were reduced following treatment with apomorphine [19], reflecting improvements in liver pathology.

Notably, while “PC(16:0/22:6);O1” and “TG(16:0/18:1/18:2);O1”—representative oxidized lipids—did not show significant changes post-treatment, alterations in LPLs and other fatty acids were notable. This discrepancy indicates that while some oxidized lipids may not directly correlate with disease pathology, the reduction in LPLs and fatty acids could indicate therapeutic benefits. It has been reported that oxidative stress can lead to an increase in lysophosphatidylcholine (LPC), which aligns with our findings, further supporting the role of oxidative stress in the elevation of LPLs. Lysophosphatidic acid (LPA) and lysophosphatidylinositol (LPI), intermediates in phospholipid metabolism and known GPCR activators, along with LPCs generated through oxidative stress, are thought to contribute to cellular damage and progression of liver pathology [38,39,40,41,42]. Additionally, vitamin E, a known antioxidant, has been successfully used as a therapeutic agent for MASH, due to its ability to reduce oxidative stress. This suggests that other antioxidants could similarly be effective in treating MASH by mitigating oxidative damage.

Furthermore, PTEN deficiency leads to increased oxidative stress and lipid dysregulation, contributing to the development of steatohepatitis and hepatocellular carcinoma [17]. Loss of PTEN function enhances lipid accumulation and oxidative damage in hepatocytes, which may explain elevated LPL levels observed in our model. Apomorphine’s known antioxidant properties, coupled with its ability to reduce oxidative stress and lipid peroxidation, may contribute to these observed benefits.

These findings support the hypothesis that reducing specific harmful metabolites like LPLs can mitigate liver damage and provide a therapeutic pathway for MASH. By extending these observations, we seek to better understand the function of these metabolites in the pathogenesis of human MASH. Additionally, the dopamine receptor agonist activity of apomorphine may have contributed to unexpected adverse events. Therefore, we are currently investigating the development of apomorphine derivatives that lack dopamine receptor agonist activity, with the goal of exploring their potential as a safer and more effective treatment for MASH.

In this study, while we demonstrated apomorphine’s therapeutic effects in the pathological (MASH-affected) model, we acknowledge the limitation of not including a non-pathological control group treated with apomorphine. Future studies will be needed to investigate baseline pharmacological effects of apomorphine in a non-pathological context. These follow-up studies will help clarify whether the observed metabolic changes are specific to the pathological condition or reflect a broader pharmacological action.

## 5. Conclusions

This study utilized non-targeted and targeted metabolomics to examine metabolic changes in hepatocyte-specific PTEN knockout mice, a model for MASH, identifying LPLs as key metabolites that were elevated in the pathological state and significantly reduced following apomorphine treatment, suggesting a central role for LPLs in the progression of MASH. Apomorphine’s ability to modulate these metabolic disruptions highlights its potential as a therapeutic option for MASH.

Overall, these findings enhance our understanding of metabolic pathways involved in MASH, and support the potential of LPLs as therapeutic targets in the treatment of liver diseases.

## Data Availability

Mass spectrometry data have been deposited in Mendeley Data, doi: 10.17632/ghxfg2498b.2. Any additional information required to reanalyze data reported in this paper is available from the senior author upon request.

## Supplementary Materials

The following supporting information can be downloaded at: https://www.mdpi.com/article/10.3390/antiox13111293/s1. Table S1: Summary of the MRM parameters and peak retention time.
