# Supplementary material for: Non-Targeted Metabolomics Reveal Apomorphine’s Therapeutic Effects and Lysophospholipid Alterations in Steatohepatitis
Source: Antioxidants (Basel). 2024 Oct 25;13(11):1293. doi: 10.3390/antiox13111293 (PMC11591194; doi:10.3390/antiox13111293)
Supplement: Supplementary file 1 [file antioxidants-13-01293-s001.zip › antioxidants-3226424-supplementary.pdf]

Supplementary Table S1. Summary of MRM parameters and retention times for the targeted compounds.

**Analyte**	**Precursor**	**Product**	**Collison (V)**	**Retention time (min)**
PC(16:0/22:6);O1 [M+H]+	822.6	184.1	−25	4.8
TG(16:0/18:1/18:2);O1 [M+NH4]+	890.8	577.5	−30	10.4
LPC(20:4) [M−HCOO]−	588.3	303.2	25	2.8
LPE(18:0) [M−H] −	480.3	283.3	25	3.6
LPE(20:4) [M−H] −	500.3	303.2	25	2.9
LPI(20:4) [M−H] −	619.3	303.2	25	2.4
DG(18:1/18:1) [M+NH4]+	638.6	339.3	−25	7.2
PC(16:0/22:6) [M+H]+	806.6	184.1	−25	5.4
TG(16:0/18:1/18:2) [M+NH4]+	874.8	577.5	−30	12.1
